# Mechanical performance of experimental acrylic resins modified by nanoparticles after chemical and mechanical degradation

**DOI:** 10.4317/jced.57265

**Published:** 2020-12-01

**Authors:** Luciana Machado-Santos, Nicolaos Silikas, Kusai Baroudi, Mario-Alexandre-Coelho Sinhoreti, William-Cunha Brandt, Priscila-Christiane-Suzy Liporoni

**Affiliations:** 1 Department of Restorative Dentistry, School of Dentistry, University of Taubaté, Taubaté, Brazil; 2 Dentistry, School of Medical Sciences, University of Manchester, Manchester M13 9PL, UK; 3 Postgraduate Program, School of Dentistry, University of Taubaté, Taubaté, Brazil; 4 Department of Dental Materials, Piracicaba Dental School, State University; 5 Department of Dentistry, University of Santo Amaro, Sao Paulo, Brazil

## Abstract

**Background:**

Different materials have been incorporated into the polymethylmethacrylate matrix to improve its performance. The aim of this study was to evaluate the degree of conversion (DC), the flexural strength (FS), the elasticity modulus (EM), and the effect of exposure to food-simulating liquids prior to brushing simulation on the gloss loss (GL) of experimental acrylic resins modified by nanoparticles.

**Material and Methods:**

Three different types of nanoparticles; silicon oxide (SiO2), cerium oxide (CeO2) and titanium oxide (TiO2) were added to a poly (methylmethacrylate) matrix, in proportions of 0.5wt%, 1wt% and 3wt% each, forming nine experimental groups. The acrylic resin was also tested as a control group. DC was investigated using Fourier transform infrared spectroscopy (FTIR). A three-point bending test was used for FS and EM. GL after chemical degradation and simulated brushing was evaluated using a glossmeter. Data were submitted to one and two-way ANOVA followed by Duncan’s post hoc test (α=0.05).

**Results:**

All nanoparticle-modified groups showed higher values of DC. Ce1% showed higher values of FS and EM. All other groups showed similar or lower physical-mechanical properties (FS, EM, GL). Regarding type and wt%, CeO2 and TiO2 groups had better performances and were similar to each other.

**Conclusions:**

Incorporating metal nanoparticles, especially CeO2, could improve the physical properties of the dental materials.

** Key words:**Polymethylmetacrylate, degree of conversion, flexural strength, elasticity modulus, gloss loss.

## Introduction

The widespread use of dental resins comes from their ability to take complex shapes and to be stable under heating and pressure (1). Dental resins are, in general, methacrylate-based due to its biological, physical, chemical, mechanical and aesthetic properties. The poly (methylmethacrylate) is the most frequently used material for the manufacturing of dental and alloplastic prostheses to repair craniofacial deformities (1,2).

In the late 1930s, heat-polymerized acrylic resins were introduced for use in denture bases (3). In the following years, microwave polymerization was introduced by Nishii (4) (1968) and has become a popular alternative to the conventional water bath method.

PMMA-based dental polymers present some favorable characteristics, such as good biological properties—they are tasteless, insoluble and compatible with oral tissues—and some favorable physical properties, including dimensional stability, resilience and resistance, yet they are easy to handle and low in cost. In clinical practice, however, many of the properties of the acrylic resins have shown flaws. A low degree of conversion after inadequate polymerization means an increased number of free radicals and therefore compromised physical properties (5). This incomplete conversion of monomers can also generate unreacted residual monomers, free in the oral environment, which in certain concentrations present cytotoxicity (6). Shibata *et al.* (7) reported that the degree of conversion of acrylic resin may be affected after the incorporation of another material, which would also lead to an increase in the amount of residual monomers.

The flexural strength of acrylic resin, also called the modulus of rupture, is the property that manifests every time the prosthesis subjected to cyclic deformation. It represents the highest stress experienced in this material at the time of rupture. A low ability to withstand such deformation under load force is the most responsible factor for the clinical incidence of premature fracture of the prosthesis (8). Clinical fracture of this material can be caused by low resistance to impact or flexural loads (9). 

Finishing and polishing should establish a smooth, glossy surface texture with optimum contour, minimizing the adhesion of biofilm and staining. The resistance of the polishing procedure, continuously affected by different medias with different pH levels in the oral environment, might have an effect on the physical properties of the restorative materials and might increase the risk of premature failures (10,11).

Different materials have been incorporated into the poly (methylmethacrylate) matrix to improve its performance (12,13); however, there is still a need for more researches in literature concerning the incorporation of filler particles in nanometric scale. Nevertheless, when considering the use of this technique of incorporating nanoparticles into other polymeric materials, the inorganic filler as reinforcement for acrylic resin colud be a promising approach.

The objective of this study was to analyze different contents of inorganic nanoparticles, consisting of silicon oxide (SiO2), cerium oxide (CeO2) and titanium oxide (TiO2), and their effects on the physicochemical properties of experimental acrylic resins. The null hypothesis was that the properties tested would not be affected by the incorporation of nanofillers to the PMMA-based resin.

## Material and Methods

-Resin and sample preparation:

Ten experimental resin formulations were tested in this study. The resin matrix consisted of poly (methylmethacrylate) (Acron MC – GC Europe, Leuven, Belgium) in microwave curable, commercially available powder (containing PMMA prepolymer, initiator and other additives) and liquid (containing MMA monomer and cross-linking agent) forms. The nanoparticles consisted of silicon dioxide (15nm), cerium oxide (25nm) and titanium dioxide (50nm) and were incorporated to the polymer powder in increasing concentrations of 0.5%, 1% and 3%wt. The nanoparticles were supplied with their surface coated with silane (MK Nano, Canada). The dispersion process within the powder was carried out in a dual asymmetric centrifugal mixer (DAC Speedmix 150.1, 50/60Hz, 3,500rpm, Germany) in a 2,000rpm cycle for 3 minutes. Then the liquid was added according to the proportion powder/liquid 2:1 and submitted to a 500rpm cycle for 1 minute. Flasks (GC Acron FRP, Leuven, Belgium) were previously prepared. Bar-shaped metal patterns were used for the FS and EM tests, and composite resin discs were used as patterns for DC and GL. These patterns were invested individually in laboratory silicone (ZetaLabor Titanium, Zhermack, Badia Polesine, Italy) and then covered again with silicon and type III dental stone (Scola-cast powder; Scola, Cheshire, England). The patterns were removed, and the experimental acrylic resin was packed into the silicone mold at its dough stage. Flasks were closed and slowly and gradually pressed in a hydraulic press until establishing 1,25Kg. After the pressing time, the flasks were placed in a microwave oven (Daewoo KOG-6L6B, Daewoo Electronics, England) and underwent the polymerization process for 10 minutes, as follows: phase 1 – 3 min/40% potency; phase 2 – 4 min/0% potency; and phase 3 – 3 min/90% potency. Next, the flasks were bench-cooled at room temperature. The samples were finished with an electric mini drill (Dremel 3000, 3,000-5,000rpm, Robert Bosch Tool Corp., USA) accordingly to the test to be performed. Only samples used for surface gloss had one side polished, using 600-1,200 grid abrasive sandpaper (Silicon carbide, Jiansha Brand, Jiangsu, China). All samples were dry stored at 24°C±1°C for 24±2h until tests.

-Degree of Conversion:

For each experimental group, ten polymerized samples (n=10) with 10mm diameter and 2mm thickness were tested to assess the degree of monomer conversion by FTIR. A sample of the experimental uncured resin was also analyzed as a negative control. The measurements were made in absorbance with the FTIR spectrometer (Avatar 360 FT.IR, Thermo Electron Co., Madison, WI, USA) operating under the following conditions: wavelength between 300 and 4,000 cm-1, resolution of 4 cm-1 and 32 scans. The degree of conversion was determined by the following equation.

DC (%) = 100 × [1 − (R polymerized/R nonpolymerized)], where R represents the ratio between the absorbance peak at 1,638cm−1 and 1,720cm−1.

-Flexural Strength and Elastic Modulus:

One hundred samples (n=10), 65X10X4mm, were prepared for the three-point bending test. The flexural strength and modulus were measured using a universal testing machine (Zwick / Roell Z020, Zwick Testing Machines, England)). The samples were placed on supports 50mm apart. A load of 500N, with a speed of 5mm/min-1 was applied to the center of the sample until fracture occurred. The flexural strength was calculated using the FS=3Fl/(2bh2), where F is the maximum applied load, l is the length between supports (50mm), b is the sample width and h is the thickness. The elastic modulus was calculated using the formula EM=Fl3/(4bh3d), where d is the deflection.

-Surface Gloss Loss after Chemical and Mechanical Degradation:

Sixteen disc-shaped samples from each composite, 10X2mm, were randomly distributed into four groups (n=4) according to the respective solution in which they were immersed: AS – Artificial Saliva, He – Heptane P.A., CA – Citric Acid 0.02M and Et – Ethanol 50%. He, CA and Et are the mediums recommended by the FDA (1976) to be used as food-simulating liquids (11). Their respective compositions and the types of food they represent are described in Table 1. Samples were stored for seven days in their respective solutions. After this period, samples were ultrasonically cleaned to remove residuals. After the exposure to the chemical solutions, samples were submitted to 18,000 brushing cycles under a vertical load of 200g, simulating a period of approximately eighteen months of toothbrushing. Toothbrushes with compact heads and soft nylon bristles were adapted in the toothbrushing-simulating machine. This apparatus provided linear toothbrushing movements across the samples at a speed of 250 cycles per min, with a double pass of the brush head over the surface.

**Table 1:**
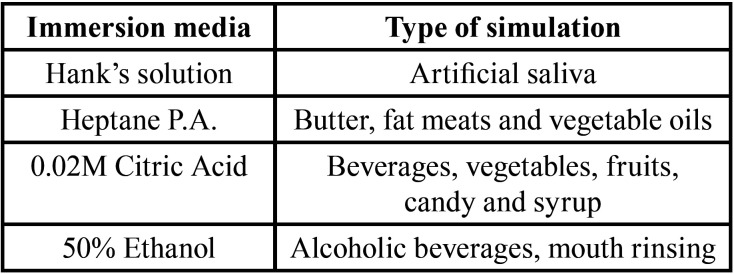
Food-simulating liquids used.

Three measurements were conducted on the top surface of the acrylic resin samples, in two moments: after the polishing procedure (baseline) and after the end of mechanical toothbrushing. Gloss was measured using a small-area glossmeter (Novo-curve, Rhopoint Instrumentation, East Sussex, UK), with a square measurement area of 2X2mm and 60° geometry. Gloss measurements were expressed in gloss units (GU), taking the average of the three measurements. A 10-mm-thick black polytetrafluoroethylene mold was placed over the samples during measurements to enable accurate sample positioning and eliminate the influence of the overhead light.

-Statistical Analysis:

The data were analyzed by one and two-way ANOVA followed by Duncan post hoc test. Statistical significance was established at α=0.05 for all tests.

## Results

The mean and standard deviation values of DC, FS and EM are reported in Table 2. Table 2 shows that the DC means for group 1 are significantly lower than all other groups; group 10 had the second-lowest average and was also significantly lower than those of other groups; and groups 4 and 8 did not differ significantly but showed averages significantly lower than the average of group 6.

**Table 2:**
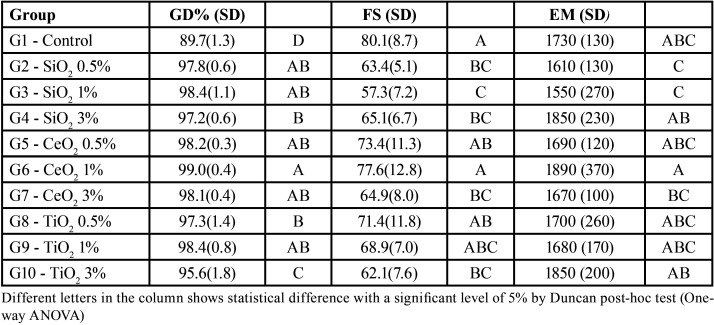
Means and Standard Deviations for DC (%), FS (MPa) and EM (MPa).

In assessing the influence of nanoparticle type and weight percentage on DC (excluding Group 1), there was a statistically significant influence of the two factors, independently. The variance analysis showed that there was no significant interaction between the two factors studied. Based on multiple comparisons regardless of the percentage of the particle weight factor, the acrylic resin with the addition of titania presents significantly lower means of degree of conversion than the other evaluated acrylic resins. Furthermore, the acrylic resin with the addition of silica presented values of DC significantly lower than with ceria. Regardless of the nanoparticle type factor, the material with 3% of particles had, on average, a significantly lower DC than those seen at other levels of the factor type nanoparticles; however, materials with 0.5% and 1% particles did not differ from each other.

For FS, as shown in Table 2, the mean in Group 3 is significantly lower than the average flexural strength of groups 8, 5, 6 and 1. In addition, the means of Groups 10, 2, 7 and 4 were not statistically different among themselves (p˃0.05), but the means of these groups were statistically lower than those for groups 6 and 1. The elasticity modulus analysis showed that the means for group 3 and 2 are significantly lower than the means of groups 10, 4 and 6, and the values of group 7 were significantly lower than the means obtained for group 6.

Table 3 shows comparisons of FS and EM according to the type and percentage by weight of nanoparticles added to the acrylic resin. When evaluating the influence of nanoparticle type and weight percentage in the FS and EM (excluding group 1), it was observed that interaction between the two factors had a statistically significant effect. According to multiple comparisons, for the acrylic resin with 0.5% of nanoparticles, the mean of FS of the material with silica was significantly lower than in materials with ceria or titanium oxide; moreover, there was no statistically significant difference (*p*<0.05) in means of FS between materials with ceria and titanium oxide. In the acrylic resin with 1% of particles, the results showed that there was a statistically significant difference between the three materials tested, where the mean of FS of the material with silica was significantly higher than the other two materials.

**Table 3:**
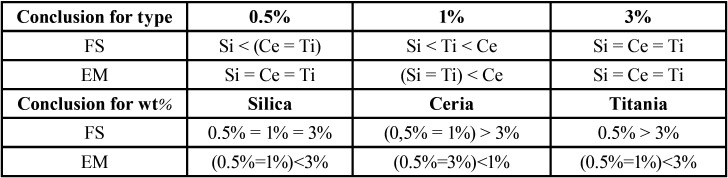
Group comparisons for FS and EM, according to nanoparticle type and % by weight. (Two-way ANOVA, Duncan test, *p*<0.05).

Furthemore, the mean of FS of titania was significantly higher than the mean of ceria. In acrylic resins with 3% of nanoparticles, there was no statistically significant difference in the means of FS among the 3 types of material. Ceria showed no significant difference in the mean of FS between 0.5% and 1%; however, 3% had significantly higher values. In the case of the material with titania, the mean of FS of the material with 0.5% of nanoparticles was significantly higher than 3%. Considering measurements of EM, the acrylic resin with 0.5% or 3% of nanoparticles showed no statistically significant difference among 3 types of added material. However, in relation to the 1% nanoparticles, the results showed that the means of EM of the material with silica or titania did not differ significantly, although these materials have means of EM significantly lower than the material with ceria.

In the evaluations of acrylic resin added with 3 types of materials, the result for materials with silica or titania showed that the EM of the resin with 0.5% or 1% particles did not differ significantly; however, these groups had significantly lower elasticity than the resin with 3% of nanoparticles. Finally, acrylic resins added with ceria 0.5% or 3% had means of EM similar to each other and significantly lower than 1%.

For GL percentage, there was no significant interaction between the two factors (group and immersion media), so analysis of variance showed that each evaluated factor did not affect the results obtained in the levels of another factor. As for the multiple comparisons, regardless of the immersion media used, the means of GL percentage of groups 5, 6, 10, 9 and 7 were significantly lower than the means found in groups 4, 2 and 1. Moreover, the averages of groups 5, 6, 10, 9 and 7 did not differ significantly from each other. The same result was found for the means of groups 4, 2 and 1, which were similar to each other. Finally, regardless of the groups, there is no statistically significant difference between the four types of immersion media in the means of gloss loss percentage.

In assessing the influence of immersion factors, type of nanoparticle and particle weight percentage as GL percentage (excluding group 1), it was observed that two of the three factors evaluated had a statistically significant influence. In addition, there was a significant interaction between the three factors studied, whether two or three factors were combined at the same time. Furthermore, the results showed that the groups immersed in AS and CA were not statistically different, although they exhibit significantly lower means of GL percentage than the group immersed in Et. Regarding the type of nanoparticles added to the resins, regardless of the immersion media and the percentage by weight of nanoparticles added to the material, the means of GL percentages did not differ significantly among the acrylic resins with ceria and those with titania, which showed significantly lower means than materials with silica.

## Discussion

The tested hypothesis was partially validated, since the nanoparticle addition in the model dental acrylic resin allowed higher DC values and increases in gloss after mechanical and chemical degradation; however, the experimental resins presented similar or reduced physical-mechanical properties compared to the control group.

The increase in DC may be explained by a better saturation of the monomer matrix, which was enhanced by adding the silane-treated nanoparticles (14), enabling the formation of a crosslinked polymer structure. Furthermore, a shorter dough time was observed as the content of nanoparticles was increased. These findings may suggest a proper formation of the polymer structure.

The heating system used in this study was microwave energy. A regular microwave oven heats the material by passing microwave radiation through it (15). The lower DC values observed in the control group could suggest that some amount of free or trapped unreacted monomers remained. Among other reasons, premature termination may occur because of quick volatilization of reactants, caused by the intense increase in temperature. Some transition metal oxides can absorb microwave to be heated; however, SiO2 appears to absorb it weakly, exhibiting little raising in temperature and metal catalysts such as CeO2 do not have a very high susceptibility to incident microwave. The microwave dielectric heating effect uses the ability of some solid materials of absorbents or catalysts to transform electromagnetic energy into heat via their dielectric loss and thereby drive catalytic reactions (15).

So, the microwave could produce thermal energy in the silane surrounding the nanoparticles without creating heat in the nanoparticles themselves. This mechanism concerning the nanoparticles could decrease the temperature, slowing down the propagation phase of the polymerization process. Also, according to Van Noort, if the temperature reaches 100°C, before the polymerization is completed, a gaseous monomer will be formed, increasing the amount of porosity. This could explain the higher degree of conversion for all groups containing nanoparticles (16).

 According to Takabayashi (16), the ISO standard for Type 5 denture base materials require more than 65 MPa of flexural strength and a modulus of elasticity of 2000 MPa. Therefore, our results indicate that SiO2 tends to reduce FS values to a range below the acceptable level and that all groups, including the control group, presented EM values below 2000MPa. Given the function of a denture base in a removable prosthesis, high flexural strength and flexural modulus would help in resisting the torsional forces in function, which leads to a longer clinical service life for the prosthesis. In our study, the control and Ce1% groups presented higher values of FS, and Ce1% presented a slightly higher value of EM. Even though other groups presented lower values, they are still within the range determined by ISO.

In our study, therefore, Ce1% presented values statistically similar to the control group. All other experimental groups presented lower values. The latter is often seen in the literature. Many studies have tested the flexural strength and flexural modulus after incorporating some sort of reinforcement in the PMMA dental polymer, finding the following results. Paleari *et al.* (17) added 2-tert-butylaminoethyl methacrylate (TBAEMA) for antimicrobial purposes and concluded that the acrylic resin was softened by it, dramatically decreasing flexural properties. Ellakwa *et al.* (18) added AlO2 fillers to try to increase the flexural strength and thermal diffusivity, but they concluded that none of the polymers possessed the desired properties for the purpose of the material. Sodagar *et al.* (19) concluded that the incorporation of TiO2 and SiO2 nanoparticles adversely affected flexural strength, which they related to the possible agglomeration of these fillers. On the other hand, Sahin *et al.* (20) added 2-hydroxyethyl methacrylate (HEMA) and isobutyl methacrylate (IBMA) to a PMMA matrix and found that all experimental groups had higher values of flexural strength compared to the control group.

Silanization is the covering of a surface with organo-functional-chloro or alkoxysilane molecules. Oxide components like silica nanoparticles and metal oxide surfaces can be silanized because they contain hydroxyl or silanol groups which attack and displace the alkoxy groups (-OCH3) on the silane, thus forming a covalent -Si-O-Si- bond (14). The goal of silanization is to form bonds across the interface between mineral/inorganic components and organic components present in the PMMA. The silanization of the nanoparticles used in this study may have allowed them to bond with the polymeric matrix, participating in the chain formation; however, nanoparticles tend to form agglomerations (21) with voids among them, which may have created points of tension during flexural deformation. Fracture propagation seems to have been facilitated, since most groups containing nanoparticles showed lower FS values compared to the control group. Those values also tended to be lower for the higher percentage of nanoparticles.

Previous studies have assessed superficial changes in dental composites by food-simulating liquids. These alterations have been attributed to the degradation of the polymer matrix and of the resin-filler interface, and to the loss of inorganic filler particles (11). In the oral environment, PMMA-based dentures are exposed intermittently to chemical agents found in saliva, food and beverages, and continuously to acid biofilm resulting from bacterial decomposition of debris (10,11). The period of immersion chosen for this study was meant to accelerate the effect of the food-simulating liquids (11). Toothbrushing has been shown to cause superficial changes in composite materials and was applied in this study to associate mechanical degradation (22).

It might be expected that smoother surfaces would demonstrate higher gloss values. Furthermore, the gloss is material dependent and is influenced not only by the surface roughness but also by other factors such as the difference in refractive indices of the resin matrix and the fillers (23,24).

In the present study, the highest gloss loss was seen in the control group and in groups containing silica. However, except for Ti1%, significant increases in gloss values after mechanical and chemical degradation were observed in the ceria and titania groups. Unexpectedly, citric acid proved to be the immersion media that provided increase in gloss, although it was expected that such a low pH would cause the erosion of polymeric chains, loss of nanofillers and increase in roughness (10). Therefore, it might be concluded that the composition of the material rather than the roughness might have an effect on the gloss. Even though it was expected that the incorporation of nanofillers would result in more stability of the soft resin matrix and less filler plucking, leading to enhanced wear resistance (18,25), it is not possible to conclude whether there was a loss of substance.

Even though the incorporation of nanoparticles significantly increased the percentage of reacted carbon double bonds and provided gloss increase after chemical and mechanical degradation, physical properties such as flexural strength and elasticity modulus showed similar values to the control group. Reinforcement with nanoparticles seems to be a promising approach; however, further studies are necessary in order to assess their influence on diverse highly demanded mechanical properties.

## Conclusions

The study suggests that incorporating metal nanoparticles, especially CeO2, into dental materials provides a reinforcement method, with enhanced chemical characteristics, which is expected to optimize the physical properties of the material. This could result in improved restorative dental materials and craniofacial prosthesis, aggregated higher quality and durability.
